# Five additions to the list of Sepsidae Diptera for Vietnam: Perochaeta cuirassa sp. n., Perochaeta lobo sp. n., Sepsis spura sp. n., Sepsis sepsi Ozerov, 2003 and Sepsis monostigma Thompson, 1869

**DOI:** 10.3897/zookeys.70.766

**Published:** 2010-11-29

**Authors:** Yuchen Ang, Rudolf Meier

**Affiliations:** Evolutionary Biology Laboratory, Department of Biological Sciences, National University of Singapore, Singapore 117543, Republic of Singapore

**Keywords:** Sepsidae, *Perochaeta*, *Sepsis*, new species, new records, Vietnam

## Abstract

A recent collecting trip to Vietnam yielded three new species and two new records of Sepsidae (Diptera) for the country. Here we describe two new species in the species-poor genus Perochaeta (Perochaeta cuirassa **sp. n.** andPerochaeta lobo **sp. n.**) and one to the largest sepsid genus Sepsis (Sepsis spura **sp. n**.) which is also found in Sumatra and Sulawesi. Two additional Sepsis species are new records for Vietnam (Sepsis sepsi Ozerov, 2003; Sepsis monostigma Thompson, 1869). We conclude with a discussion of the distribution of Perochaeta and the three Sepsis species.

## Introduction

The Sepsidae are a moderately large, cosmopolitan family of saprophagous flies, with over 300 extant species recorded from all zoogeographic regions (Ozerov 2005). Most species are attracted to dung, carrion, and other malodorous, decaying organic substrates (Pont and Meier 2002); i.e., by using different substrates in different microhabitats, the sepsid fauna from a specific locality can be quickly explored. Separating sepsids from the remaining saprophagous insects is also relatively straightforward because most sepsids can be easily recognized based on the constriction of the first two abdominal segments which gives the flies a wasp- or ant-like habitus.

Here we update an existing species list for Vietnam by adding five species: three are new to science while two others are new records. The relatively large number of additions is due to the fact that the Vietnamese sepsid fauna remains poorly studied (e.g. Ozerov 1993, Iwasa and Thinh 2008). The current species list comprises 21 species in six genera and is based on the sepsid world catalogue (Ozerov 2005) and subsequent taxonomic research by Iwasa and Thinh (2008). We complement this list by adding five species that were collected during a brief collecting trip in July 2010: Perochaeta cuirassa sp. n., Perochaeta lobo sp. n., Sepsis spura sp. n., Sepsis sepsi and Sepsis monostigma.

## Materials and methods

All five species were collected between 11–16 July 2010 from Northern Vietnam (Ba Vi National Park and Sa Pa Valley). Cow dung was placed in various habitats for at least a few hours to attract sepsids, which were then caught by sweep-netting. Additional material for Sepsis sepsi and Sepsis spura were also collected previously in Indonesia (Sulawesi and Sumatra) in 2007 and 2009.

Specimens were photographed using a Leica Z16 APO-A stereomicroscope fitted with a DFC425 digital microscope camera, and then digitally traced to illustrations using a Wacom© PTZ 630 tablet. We also amplified and sequenced a 544-bp fragment of cytochrome oxidase c subunit I (COI) within the DNA barcoding region for the two new Perochaeta species based on the methods described in Tan et al. (2010). All type specimens and additional material are vouchered in 95% ethanol and kept in the Cryogenic Collection of the Raffles Museum of Biodiversity and Research (RMBR), National University of Singapore, Singapore. We adopt the taxonomic terminology as described by Merz and Haenni (2000) for adult morphology (excluding terminalia) and Sinclair (2000) for male genitalia.

## Taxonomy

Describing new species in genera that have not been revised recently requires extra care and justification, because the risk of creating new synonyms based on overlooked or misinterpreted species in the literature is high. Fortunately, this is not the case for Perochaeta, which has only three described species (see Ozerov 2005 and Ang et al. 2008) and no synonyms. In addition, the descriptions and illustrations for the described species are of good quality. Furthermore, molecular data are consistent with distinct species: the two new Perochaeta species are separated by 3.3% for the barcoding gene COI (uncorrected pairwise distances); while the distances of either to Perochaeta dikowi are 11.4% and 11.8% (see Table 1 for variable base pairs).

**Table 1.** Seventy-two variable base pairs in a 544-bp COI sequence fragment for three Perochaeta (Positions according to Drosophila melanogaster COI).

Base Pair No.	0	0	0	0	0	0	0	0	0	0	0	0	1	1	1	1	1	1	1	1	1	1	2	2	2	2	2	2	2	2	2	2	2	2	2	3	3	3	3	3	3	3	3	3	3	3	3	3	3	3	3	3	3	4	4	4	4	4	4	4	4	4	4	5	5	5	5	5	5	5	5	5
		4	5	5	6	6	6	7	7	8	8	9	9	2	2	3	3	3	4	4	5	7	8	0	0	2	2	3	4	4	4	5	5	6	7	7	0	0	0	0	1	1	2	2	2	2	3	6	7	8	8	8	9	9	1	1	2	2	2	4	4	5	9	9	0	1	1	2	4	5	6	7	7
		6	1	5	0	3	6	2	5	1	7	1	3	3	6	2	3	8	4	7	0	1	6	1	4	2	5	1	3	6	7	2	8	1	0	9	4	6	7	9	2	5	4	5	7	8	6	6	8	1	4	7	0	6	1	7	3	6	7	2	4	3	2	5	7	0	9	8	9	5	7	7	9
Perochaeta dikowi	T	C	T	T	A	T	T	A	G	T	N	G	T	A	T	C	T	C	C	A	A	A	A	A	A	G	A	A	C	C	A	A	A	A	T	C	T	T	A	T	T	A	T	A	C	T	T	T	T	A	C	T	T	A	T	T	C	T	T	A	T	C	T	A	A	A	C	A	T	T	C	T
Perochaeta cuirassa	C	C	A	C	T	A	A	G	C	A	T	A	C	T	A	T	C	T	T	G	T	T	G	T	G	A	T	A	T	T	A	A	C	G	A	T	A	C	T	C	C	T	C	T	T	C	T	G	C	A	G	A	T	T	A	A	T	C	C	T	A	T	A	A	A	T	T	T	A	A	T	A
Perochaeta lobo	C	T	A	C	T	A	A	A	C	A	T	A	T	T	A	T	C	T	T	G	T	T	A	T	G	A	T	C	T	T	G	G	T	G	A	T	A	C	T	T	T	T	C	T	T	C	C	A	C	G	G	A	C	T	A	A	T	C	C	T	A	C	A	G	C	T	T	T	A	A	C	A

Describing a new Oriental Sepsis species in the absence of a generic revision is more problematic given that the genus is the largest in Sepsidae (ca. 80 described, valid species). Of the 23 Sepsis recorded in the Oriental region (Ozerov 2005), the Sepsis species described here closely resembles the widespread Sepsis nitens Wiedemann, 1824 which has two synonyms (Sepsis brevicosta Brunetti, 1910 and Sepsis tuberculata Duda, 1926). Duda’s (1926) description and fore leg illustration of Sepsis tuberculata are sufficiently detailed to confirm that it is indeed a synonym of Sepsis nitens. However, Brunetti’s (1910) rather vague description of Sepsis brevicosta based on one male from Calcutta and a few females from localities in other Indian localities (Calcutta, Shencotta, Tinpahar and Pusa), is more difficult to interpret. He describes Sepsis brevicosta’s fore femur as having “a small bump [on the ventromedial region] with three or four strong short spines”. This description of a “bump” is in agreement with Sepsis nitens (Fig. 21) while the ventromedial protrusion of the new species of Sepsis is shaped more like a spur (Figs 24, 26). In addition, Brunetti’s Sepsis brevicosta is known from India, while the new Sepsis species described here is only known from Vietnam (Lào Cai) and Indonesia (Sumatra and Sulawesi).

**Figures 1–9.**
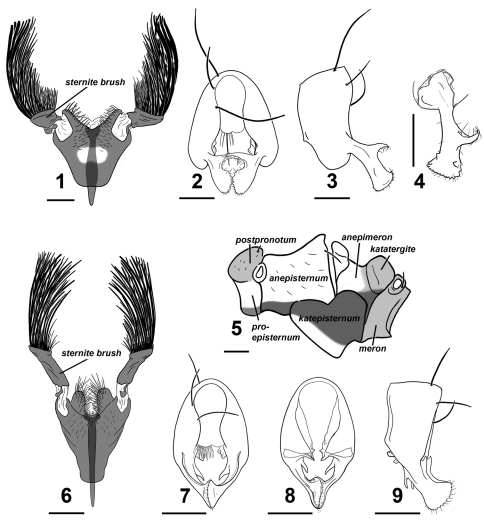
Perochaeta cuirassa and Perochaeta lobo **♂**. Perochaeta cuirassa: **1** 4^th^ sternite, ventral **2** hypopygium, dorsal **3** hypopygium, lateral **4** surstylus, lateral (inward facing) **5** pleural pruinosity pattern, lateral. Perochaeta lobo: **6** 4^th^ sternite, ventral **7** hypopygium, dorsal **8** hypopygium, ventral **9** hypopygium, lateral. Scale bars: 0.5mm.

As argued elsewhere, new species are hypotheses that are dependent on species concepts; it is therefore desirable that authors who describe species are explicit about which species concept was used and whether other species concepts would come to different conclusions (Laamanen et al. 2003, Tan et al. 2008, 2010). Here we apply the Hennigian species concept (Meier and Willmann 2000) and use morphology and DNA sequence data (for Perochaeta) to estimate the species boundaries. The two new Perochaeta species are sympatric and the new Sepsis species is parapatric with Sepsis nitens. In both cases we have not seen any intermediate specimens so that there is no evidence for hybridization. This supports our species hypotheses. However, both Perochaeta species are so rare that this test is relatively weak. As pointed out by Lim et al. (in press), in such cases descriptions should only be prepared if the species are unusually distinct. This is the case here, whereby the two new species can be clearly distinguished based on both morphology [cf. sternites and hypopygia of Perochaeta cuirassa (Figs 1–4) and Perochaeta lobo (Figs 6–9)] and DNA sequence data (Table 1). When the distributional and morphological data are applied to the remaining species concepts in Wheeler and Meier (2000), most support the same species boundaries. The only exception is Mishler and Theriot’s (2000) phylogenetic species concept that requires a phylogenetic analysis before species can be delimited. However, such analyses are currently unavailable. We now describe the new Perochaeta and Sepsis species and state the new records for the two Sepsis species.

### Perochaeta cuirassa

Ang 2010 sp. n.

urn:lsid:zoobank.org:act:76BA1CDF-D467-45D0-9898-305C21045C6D

Figs 1–5 

#### Material.

*Holotype.* ♂ (RMBR), **Vietnam**, Lào Cai Province, Sa Pa Valley. Baited with cow dung at forest edge next to a small cascade alongside highway, ca. 850m along the road westward of the Thác bạc (Silver Waterfall) tourist attraction [22°23'23.90"N; 103°44'50.32"E, elevation 2600m above sea level, ASL]. Collected 16.VII.2010 (Ang Y). *Paratypes*. 2 ♂ (RMBR), collected from same locality and time as holotype.

#### Etymology.

The specific epithet refers to the shape of the main scleral plate for the 4^th^ sternite, which resembles a cuirass or breastplate armor.

#### Diagnosis.

**Figures 10–14.**
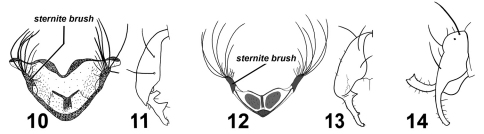
Various Perochaeta 4^th^ sternites and hypopygia.Perochaeta hennigi ♂ redrawn from Ozerov (1992): **10** 4^th^ sternite, ventral **11** hypopygium (half), dorsal. Perochaeta dikowi ♂ redrawn from Ang et al. (2008): **12** 4^th^ sternite, ventral **13** hypopygium (half), dorsal. Perochaeta orientalis ♂ redrawn from Duda (1926): **14** hypopygium, lateral.

Adult male Perochaeta cuirassa is very similar to Perochaeta lobo and can only be reliably distinguished from the latter based on the 4^th^ sternite [cf. Perochaeta cuirassa (Fig. 1) and Sepsis lobo (Fig. 6)]: The sternite in Perochaeta cuirassa lacks distinct lobes on the posterior end of the 4^th^ sternite, while the sternite brush is thick and squat (as opposed to long and thin in Perochaeta lobo), and the main scleral plate is much broader (long as wide) than in Perochaeta lobo (twice long as wide). The hypopygium [cf. Perochaeta cuirassa (Figs 2–4) and Perochaeta lobo (Figs 7–9)] is also distinct, with Perochaeta cuirassa bearing a large median, decussating protrusion on the dorsal side of the surstylus, while Perochaeta lobo has a sub-median protrusion on the ventral side of the surstylus. Perochaeta cuirassa is also readily distinguished from all other Perochaeta species based on the morphology of the 4^th^ sternite and hypopygium: The sternites brush of Perochaeta cuirassa (Fig. 1) has significantly more bristles (>40 per brush) than either Perochaeta hennigi Ozerov, 1992 (Fig. 10) or Perochaeta dikowi (Fig. 12), both of which have only 5–6 large bristles in addition to a few weaker bristles. Perochaeta cuirassa also has strong bristles lining the distal margin of the sternite, which are not found in Perochaeta dikowi or Perochaeta hennigi. The surstylus of Perochaeta cuirassa (Fig. 2) resembles that of Perochaeta hennigi (Fig. 11), but can be distinguished by the large median surstylus projection, which is long and curved in Perochaeta cuirassa but short and broadly triangular in Perochaeta hennigi. Both Perochaeta dikowi (Fig. 13) and Perochaeta orientalis (De Meijere, 1913) (Fig. 14) lack large median projections. Perochaeta cuirassa can further be distinguished by the radial-medial cross-vein dividing the discal-medial cell which is in a ratio of 3 : 1 in Perochaeta cuirassa, 2.5 : 1 in Perochaeta dikowi, 2 : 1 in  Perochaeta hennigi and 1 : 1 in Perochaeta orientalis.

#### Description (male).

##### Colour.

Head capsule mostly black except for thin yellow strip along subgena and parafacial area. Lunule, facial carina and antennae light brown; antennal groove dark brown. Proboscis brown. Thorax, scutellum and abdomen wholly black. Legs largely yellow except for the following: basal regions of fore coxa brown, mid and rear femora with a dark half-ring subapically (edges of which are diffuse on the apical edge), basal half of mid and rear tibiae dark brown. All tarsi with tarsomeres 3–5 brown; tarsomeres 1–2 yellow with brown region apically. Wing clear except for basicostal cell and basal third of costal cell, which is brown. Veins dark brown. Calypter creamy, margin and fringe-hairs yellowish. Haltere milky yellow with brown base.

##### Head.

Roundish, facial carina short and shallow, facial area receding. Gena and parafacial region narrow. Ocellar prominence and occipital region lightly microtomentose. Chaetotaxy: 1 *ocellar*, 1 divergent *postocellar*, 1 *outer vertical*; *inner vertical* absent. *Orbital* very reduced to absent. 2 *vibrissae.* 3–4 *postocular.* Lower fascial margin lined with setulae.

##### Thorax.

Scutum, postpronotum, scutellum and subscutellum wholly microtomentose. Mediotergite microtomentose but glossy in the medial region. Scutellum twice wide as long. Pleural pruinosity pattern (Fig. 5): Proepisternum glossy with ventral region microtomentose. Anepisternum largely glossy with anterioventral region densely microtomentose. Katepisternum largely with dense tomentosity except for glossy anterioventral region. Anterior side of anepimeron glossy while posterior side lightly microtomentose and posterioventral region densely microtomentose. Postpronotum, katatergite, meron and metepimeron lightly-dusted. Chaetotaxy: 1 *apical scutellar*, 1 reduced, setulae-like *basal scutellar,* 1 *dorsocentral*, 1 *postalar*, 1 *supraalar*, 2 *notopleural*, 1 *postpronotal*, 1 *anepisternal* and 1 *posterior spiracular*. Postpronotoum, prescutum and anepisternum with few, sporadic setulae.

##### Legs.

Forelegs unmodified in males; all femora and tibiae without posteriodorsalor anteriodorsal setae. Mid tibia with row of short setae on anterior apex. Rear tibia with barely-visible osomoterial patch on medial posteriodorsal side. Rear basitarsus with three ventral dark spines basally.

##### Wings.

Without pterostigma. Veins bare. Covered with microtrichiae except for costal, subcostal, basal-medial and posterior-cubital cells, as well as base of cells r1 and r2+3. Microtrichiae sparse on basal region of basal-radial and discal-medial cells. Radial-medial cross-vein divides discal-medial cell by ratio of 3 : 1. Length: 4.6–4.9 mm.

##### Abdomen.

Glossy black; syntergite 1+2 – tergite 5 normal, tergite 6 missing, syntergite 7+8 present and extending ventrad as a narrow sclerite. Spiracles 1–4 on intersegmental membrane, spiracle 5 on ventral margin of tergite 5, spiracle 7 and 8 adjacent on margin of syntergite 7+8. Sternite 1 broadly rectangular while sternite 2 is triangular, tapering posteriorly; sternite 3 is oblong. 4^th^ sternite heavily modified (Fig. 1); greatly expanded, long as is wide, and in the shape of a breastplate. Two stout moveable appendages (= sternite brushes) branch off laterally; each appendage resembles a painter’s brush, with large, thick bristles on the outer side and shorter, thinner bristles on the inner side.Posterior edge of 4^th^ sternite invaginated with strong setae lining the outer discal margin as well as submedially. A Y-shaped apodeme extends underneath and anterior to the 4^th^ sternite.

##### Hypopygium

(Figs 2–4). Cercal plate with two very weak lobes; distal margin of each lobe covered with numerous setae. Hypopygium triangular with a two tooth-like projections on the inner side basal to where the surstylus branches off (Fig. 4). Surstylus itself fused to hypopygium, angled dorsally, and branches off subterminally (Fig. 3). Each surstylus has a large hook-like median projection that curves dorsally and decussates. Terminal section of surstylus shaped like a scapula, with cuticular “teeth” and setulae on distal margin, and a small inward-facing “tooth” on both the ventral and dorsal region subterminally pointing towards the median.

#### Distribution.

Vietnam (Lào Cai).

### Perochaeta lobo

Ang 2010 sp. n.

urn:lsid:zoobank.org:act:5B7C6CFA-9DCC-4377-9893-CBD789E333E9

Figs 6–9 

#### Material.

*Holotype.* ♂ (RMBR), **Vietnam,** Lào Cai Province, Sa Pa Valley. Baited with cow dung at forest edge next to a small cascade alongside highway, ca. 850m along the road westward of the Thác bạc (Silver Waterfall) tourist attraction [22°23'23.90N; 103°44'50.32E, elevation 2600m above sea level, ASL]. Collected 16.VII.2010 (Ang Y).

#### Etymology.

The specific epithet is a phonetic translation of Greek “λοβό”, which refers to the large, distinct lobe found on each lateral half on the posterior margin of the 4^th^ sternite.

#### Diagnosis.

The adult male is very similar to Perochaeta cuirassa and can only be reliably distinguished based on the 4^th^ sternite and hypopygium. The 4^th^ sternite [cf. Perochaeta lobo (Fig. 6) and Perochaeta cuirassa (Fig. 1)] can be distinguished to species by the presence of distinct lobes on the posterior end, the long, thin sternite brush (as opposed to short and squat in Perochaeta cuirassa), and the sternite itself being narrower (half wide as long). The structure of the hypopygium (Figs 7–9) is also diagnostic given that it is the only Perochaeta with a surstylus that has a dorsal flap along its length and a long distal-pointing projection sub-basally. Other diagnostic characters that distinguish Perochaeta lobo from Perochaeta dikowi, Perochaeta hennigi and Perochaeta orientalis as described in diagnosis for Perochaeta cuirassa.

#### Description (male).

##### Colour.

As described in Perochaeta cuirassa except for fore and rear basitarsi, which are brown with yellow base, mid basitarsus yellow with slight brown region apically.

##### Head.

As described in Perochaeta cuirassa.

##### Thorax.

As described in Perochaeta cuirassa; pleural pruinosity patternas in Fig. 5.

##### Legs.

Forelegs unmodified; as described in Perochaeta cuirassa.

##### Wings.

Coloration, venation and microtrichia distribution as described in Perochaeta cuirassa. Length: 4.2 mm.

##### Abdomen.

Tergites and sternites 1–3 as described in Perochaeta cuirassa. Sternite 4 heavily modified (Fig. 6); almost twice long as wide and raised from the abdomen. Posterior edge of 4^th^ sternite deeply invaginated and raised to form two large distinct lobes densely populated with strong bristles, mainly lining the outer discal margin. Two long, thin moveable appendages (= sternite brushes) branch off laterally on the posterior end of the sclerite, each with large, thick bristles on the outer region and some shorter, thinner bristles closer to the inside. A Y-shaped apodeme extends underneath and anterior to the 4^th^ sternite.

##### Hypopygium

(Figs 7–9). Cercal plate with two very weak lobes; distal margin covered with numerous setae. Hypopygium triangucuspid projection before the base of the surstylus. Surstylus itself fused to hypopygium and branches off terminally, with a dorsal flap along its length and a longish distal-pointing projection sub-basally. Terminal section of surstylus shaped like a scapula, with distal-pointing cuticular “teeth” and setulae on distal margin, and a very small inward-facing “tooth” on both the ventral and dorsal region subterminally pointing towards the median.

#### Distribution.

Vietnam (Lào Cai).

### Sepsis monostigma

Thompson, 1869

Figs 15–17

Sepsis monostigma  Thompson, 1869. Kongliga svenska fregatten Eugenies resa omkring Jorden, 2(1): 443.

#### Material.

♂♂♀♀ (RMBR), **Vietnam,** Lào Cai Province, Sa Pa Valley. From ex culture established from ♀ collected from dung on cow farm, 4km NE of Sa Pa town [22°21'28.19"N; 103°51'53.35"E, elevation 1250m ASL]. Collected 15.VII.2010 (Ang Y).

**Figures 15–23.**
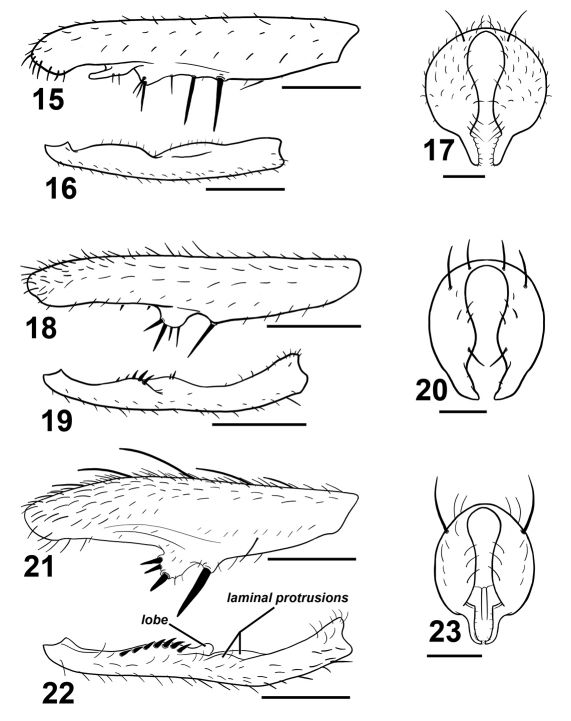
Various Sepsis forelegs and hypopygia. Sepsis monostigma:**15** fore femur, posterior **16** fore tibia, posterior **17** hypopygium, dorsal. Sepsis sepsi: **18** fore femur, posterior **19** fore tibia, posterior **20** hypopygium, dorsal. Sepsis nitens: **21** fore femur, posterior **22** fore tibia, posterior **23** hypopygium, dorsal. Scale bars: 0.5mm.

#### Taxonomic remarks.

Sepsis monostigma is an Oriental species that resembles Sepsis pseudomonostigma Ursu, 1969 but is geographically exclusive from Sepsis pseudomonostigma (which has only been recorded in South and South-east Europe and Central Asia). Sepsis monostigma can be differentiated by the two long medioventral spines (one short spine in Sepsis pseudomonostigma) on the fore femur (Fig. 15), lack of ventromedial spinules on the fore tibia (Fig. 16) and surstylus (Fig. 17) being much thinner than that in Sepsis pseudomonostimga.

#### Distribution.

China (Guandong), Taiwan, India (Jammu and Kashmir, Uttar Pradesh, West Bengal), Japan (Hokkaido Is., Honshu Is., Kyushu Is.), Korea, Philippines (Luzon Is.), Sri Lanka, South Russia (Amurskaya Oblast’, Irkutskaya Oblast’, Khabarovskiy Kray, Krasnoyarskiy Kray, Novosibirskaya Oblast’, Primorskiy Kray, Sakhalinskaya Oblast’), Vietnam (Lào Cai).

### Sepsis sepsi

Ozerov, 2003

Figs 18–20

Sepsis sepsi  Ozerov 2003. Zoologicheskiy zhurnal, 82, 10: 1276.

#### Material.

♂♂♀♀ (RMBR), **Vietnam**, Ha Tay Province, Ba Vi National Park. From ex culture established from ♀ collected from rubbish dump near temple at summit of mountain [21°3'45.84"N; 105°21'57.63"E, elevation 800m ASL]. Collected 11.VII.2010 (Ang Y). 2 ♂ (RMBR), **Indonesia**, West Sumatra, Bukit Tingei Regency, Tanjung Mutiara Dist., Bantar Gadang Beach, [0°24.792"S; 99°56.307"E 0m ASL]. Collected 6.VII.2007 (Lohman D).

#### Taxonomic remarks.

Sepsis sepsi bears some resemblance to Sepsis nitens and was initially thought to be such by Hennig but later identified it as a ‘Sepsis n.sp.’ (1941), and was formally described by Ozerov (2003). Sepsis sepsi can be distinguished from Sepsis nitens based on the row of four large spines on a large rounded ventromedial bump of the fore femur (Fig. 18) as opposed to three spines arranged triangularly on a slightly proclinate bump in Sepsis nitens (Fig. 21). The fore tibia (Fig. 19) lacks a rounded lobe present in Sepsis nitens (Fig. 22), and the surstylus differs in structure [cf. Sepsis sepsi (Fig. 20) and Sepsis nitens (Fig. 23)].

#### Distribution.

Indonesia (Sumatra, Sumbawa), Vietnam (Ha Tay).

### Sepsis spura

Ang 2010 sp. n.

urn:lsid:zoobank.org:act:581DEFE8-8274-4365-BCDF-7F34BB59A258

Figs 24–31 

#### Material.

*Holotype*. ♂ (RMBR), **Vietnam,** Lào Cai Province, Sa Pa Valley. Collected from dung on cow farm, 4km NE of Sa Pa town [22°21'28.19"N; 103°51'53.35"E, elevation 1250m ASL]. Collected 15.VII.2010 (Ang Y). *Paratypes.* 2 ♂ (RMBR), **Indonesia**, West Sumatra, Bukit Tingei Regency, Tanjung Mutiara Dist., Bantar Gadang Beach, [0°24.792"S; 99°56.307"E 0m ASL]. Collected 6.VII.2007 (Lohman D). ♂ (RMBR), **Indonesia**, N. Sulawesi, Tondano Province, Kampung Jawa [1°17'18.11"N, 124°52'30.05"E, elevation 650m ASL]. Collected 12.V.2009 (Ang Y).

**Figures 24–31.**
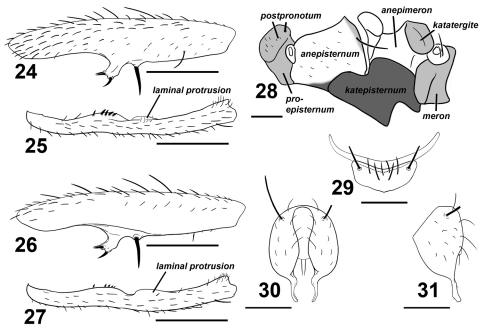
Sepsis spura. **24** fore femur, posterior **25** fore tibia, posterior **26** fore femur, anterior **27** fore tibia, anterior **28** pleural pruinosity pattern, lateral **29** 4^th^ and 5_th_ sternites, ventral **30** hypopygium, dorsal **31** hypopygium, lateral. Scale bars: 0.5mm.

#### Etymology.

The specific epithet old English for “spur”, and refers to the distinct spur-like medioventral tubercle found on the male fore femur.

#### Diagnosis.

Adult males of Sepsis spura closely resemble Sepsis nitens but can be distinguished by the following characters: (1) Medioventral tubercle on male fore femur of Sepsis spura is spur-like and bent at a forward angle with two smaller adjacent spines dorsally positioned at the end of the tubercle and one larger spine at the ventral end (Figs 24, 26), while the tubercle in Sepsis nitens is thicker on the base and has its three spines positioned more in a anterio-posterior fashion (Fig. 21). (2) The basal lamina-like projection on the fore tibia of Sepsis spura (Figs 25, 27) merges back with the tibia gently, but ends off with a distinct lobe in Sepsis nitens (Fig. 22). The short spines found posteriorly on the projection are also much weaker than those found in Sepsis nitens. (3) Sepsis spura (Figs 25, 27) has only one anterior lamina-like protrusion on the distal portion of the fore tibia, while Sepsis nitens (Fig. 22) has such protrusions on both anterior and posterior sides. (4) The surstylus of Sepsis nitens (Fig. 23) has a rather angular basal swelling and is relatively straight, curved only at the terminus, while the surstyli of Sepsis spura (Figs 30, 31) has a rounded basal swelling and is medially curved for the entirety of the surstylus. Sepsis spura can be distinguished from other Sepsis, also based on the specific structure of the male fore leg ornamentation and the shape of the surstylus.

#### Description (male).

##### Colour.

Head capsule mostly brown with a thin light brown strip on gena; fascial margin black. Vertex dark brown. Facial carina and lunule light grey-brown. Pedicel dark brown, 1_st_ flagellomere yellowish, arista brown. Proboscis whitish yellow. Forelegs wholly yellow. Mid and rear coxa yellow with brown base. Mid femur brown but yellow on basal and distal tips; mid tibia brown on basal half and diffuses to yellow on apical half. Rear femur yellow but brown on dorsal region, while rear tibia wholly brown. Fore tarsus with tarsomeres 3–5 brown, mid tarsus with tarsomeres 3–5 very lightly brown, rear tarsus with tarsomeres 4 and 5 brown. Wing clear except for basicostal cell and basal region of costal cell, which is light brown. Veins dark brown. Calypter clear, margin and fringe-hairs yellowish. Haltere white. Thorax mostly dark brown, but pronotopleuron is yellow. Abdominal tergites and sternites glossy dark brown.

##### Head.

Roundish, facial carina short and shallow, facial area receding. Gena and parafacial region narrow. Largely glossy except for lightly microtomentose occipital region. Chaetotaxy: 1 *ocellar*, 1 divergent *postocellar* (*ocellar* longer than *postocellar*), 1 *inner vertical*, 1 *outer vertical* (*outer* subequal to *inner*). *Orbital* very reduced to absent. 3–4 *vibrissae*. 3–4 *postocular.* Lower fascial margin lined with setulae.

##### Thorax.

Scutum, postpronotum and scutellum wholly microtomentose. Subscutellum microtomentose with a small glossy spot ventromedially. Mediotergite microtomentose on margins and glossy in the medial region. Scutellum twice wide as long. Pleural pruinosity pattern (Fig. 28): Proepisternum lightly microtomentose. Anepisternum largely glossy with a small strip on the anterioventral and posteriodorsal margins very lightly dusted. Balsare glossy. Katepisternum densely microtomentose. Anepimeron glossy. Katatergite, meron and metepimeron microtomentose. Chaetotaxy: 1 *apical scutellar*, 1 reduced *basal scutellar,* 1 row  *dorsocentral* with posterior-most two setae as bristles, 1 row *acrostichial* and 1 *postalar*, 1 *supraalar*, 1 *notopleural*, 1 *postpronotal*, 1 *anepisternal* and 2 *posterior spiracular*.

##### Legs.

Forelegs modified; fore femur (Figs 24, 26) with large submedian ventral spine and robust, forward curving spur-like protrusion at the median. This protrusion terminates with three short stout spines, with two adjacently positioned dorsally and one positioned ventrally. Fore tibia (Figs 25, 27) with a submedial and medial cuticular laminar extension; submedial extension with row of short spines. Additional chaetotaxy: Fore tibia with apical *anteriodorsal*. Mid femur with 1 *anterior*. Mid tibia with 2 *posterior* on median and subapex, 2 *dorsal* on basal 2/3 and apex, 1 *anterior* on apex, 1 *anterioventral* on basal 2/3 and 1 *ventral* on apex. Hind femur with 1 *anteriodorsal* on basal 2/3 and 1 *posterioventral* subapically. Hind tibia with 2 *dorsal* at median and subapically, 1 *anterior* at median and 2 *anterioventral* medially and subapically. Rear basitarsus with 2 ventral dark spines basally.

##### Wings.

Without pterostigma. Veins bare. Covered with microtrichiae except for basal half of basal-medial cell. Anterior region of r2+3 cell with sparse microtrichiae. Radial-medial cross-vein divides discal-medial cell by ratio of slightly less than 2 : 1. Length: 2.9–3.5mm.

##### Abdomen.

Tergites glossy black, syntergite 1+2 – tergite 5 normal, tergite 6 missing, syntergite 7+8 present and extending ventrad as a narrow sclerite. Spiracles 1–3 on intersegmental membrane close to tergite, spiracles 4 and 5 within respective tergites near margin. Spiracles 7 and 8 adjacent on margin of syntergite 7+8. Sternite 1 a broad rectangle with posterior invaginations while sternite 2 is triangular, tapering posteriorly; sternite 3 is oblong. Sternite 4 is V-shaped with setae and one stout discal bristle on each lateral margin; sternite 5 reduced to a thin lateral crescent (Fig. 29).

##### Hypopygium.

Cercal plate with two very weak lobes, each with single distal setae. Hypopygium and surstylus relatively setaeless. Surstylus fused to hypopygium; bulging medially at base but thin and curved medially, slightly dentate terminally (Figs 30, 31).

#### Distribution.

Indonesia (Sulawesi, Sumatra), Vietnam (Lào Cai).

### Key to species of the genus Perochaeta Duda, 1926 (males)

**Table:** 

1	Ratio of radial-medial cross-vein dividing discal-medial cell is 1:1	2
–	1:2 to 1:3	3
2	Surstylus without large medial protrusions; with small dentate terminus	Perochaeta orientalis Duda, 1926
–	Sternite brush embedded in membrane	4
3	Sternite brush as a free sclerotized lobe	5
–	Sternite brush with few bristles (ca. 5 short and 5 long bristles); surstylus with large, squat triangular protrusion on inward median	Perochaeta hennigi Ozerov, 1992
4	Surstylus without any large medial protrusions	6
–	Surstylus with large medial protrusions	7
5	Sternite brush with few bristles (ca. 5 short bristles and 5 long bristles); surstylus without large median protrusions	Perochaeta dikowi Ang et al., 2008
–	Medial protrusion emerges dorsally on surstylus; posterior margin of 4^th^ sternite V-shaped with many bristles	Perochaeta cuirassa Ang, 2010
6	Medial protrusion emerges ventrally on surstylus; posterior margin of 4^th^ sternite as two distinct lobes with many bristles	Perochaeta lobo Ang, 2010

## Discussion

### Notes on Perochaeta

Perochaeta cuirassa and Perochaeta lobo are the first Perochaeta species from Vietnam. That they are found in the same locality is surprising, given that Perochaeta is a small Oriental genus with only three described species. As discussed in Ang et al. (2008), the species in this genus appear to be only found in mid- and high-elevation localities in Asia above 650m ASL: Perochaeta dikowi was found on Fraser’s Hill, Malaysia (1300m ASL; Ang et al. 2008), Perochaeta hennigi is only known from Thawalamtenne, Sri Lanka (670m ASL; Ozerov 1992) and Tamil Nadu, India (1200m and 1400m ASL; Iwasa and Tewari 1994). The type locality of Perochaeta orientalis  is the Jiji Township in Taiwan (“Chip-chip” as designated by de Meijere; Duda 1926) which is within the Chung Yang mountain range that has an average elevation >1000m ASL, and can also found in regions such as Indonesia (Seram Is., 750m; Iwasa 2001). The new species Perochaeta cuirassa and Perochaeta lobo were found at 2600m ASL. A male Perochaeta specimen from Flores Island, Indonesia, examined by Hennig (1941) was also collected from a mid elevation site (1200m ASL). Unfortunately, the latter was poorly preserved and could not be described, but based on Hennig’s illustration, it likely constitutes a new species. Given that Perochaeta species are restricted to elevated sites, we predict that the number of species will rapidly grow as more mid and high elevation localities are sampled.

Adding two new species to Perochaeta is also of interest, because this (small) genus is very atypical for sepsids. Males of most sepsids have modified forelegs (e.g., cuticular tubercles and stout, enlarged spines; see Figs 15–16, 18–19, 21–22, 24–27) which are used to grab the base of female wings during mating (Pont and Meier 2002, Ingram et al. 2008, Puniamoorthy et al. 2008, 2009). However, Perochaeta has secondarily reduced foreleg armature, and at least Perochaeta dikowi has evolved a novel mounting behavior that does not involve the foreleg grasp (Ang et al. 2008). This has made Perochaeta a model for testing the correlation between the evolution of behavior and morphology (see Puniamoorthy et al. 2008, 2009).

### Notes on Sepsis

Sepsis spura appears to be a relatively widespread species ranging between Indonesia and Vietnam, and it can be found across all elevations, ranging from highlands (Sa Pa Valley, Vietnam; 1250m ASL) through mid elevation areas (Kampung Jawa, Indonesia; 650m ASL) to sea level (Bandar Gadang Beach, Indonesia). Such widespread species of insects are relatively common in Southeast Asia and are increasingly attracting attention because they can give rise to new species (see Balke et al. 2009). At all localities the Sepsis spura is found in low numbers on bovine dung while other Sepsis species can be very abundant. This, along with its morphological resemblance to Sepsis nitens, may explain why Sepsis spura is only described now. It is likely to belong to a clade of Sepsis species without wingspots (see Su et al. 2008).

Sepsis sepsi was first described from Sumbawa Is., Indonesia at 450m ASL (Ozerov 2003), but has subsequently been collected at low elevation sites in Sumatra as well as now at mid-elevation sites (800m ASL) in Ba Vi, Vietnam. This suggests that Sepsis sepsi is a relatively widespread species able to live in low to mid elevation habitats. We predict that it is also likely to be found in other areas between Indonesia and Vietnam. The record of the widely distributed Sepsis monostigma in Vietnam is not unexpected given that it falls within the recorded range from India to the far east of Russia.

## Supplementary Material

XML Treatment for Perochaeta cuirassa

XML Treatment for Perochaeta lobo

XML Treatment for Sepsis monostigma

XML Treatment for Sepsis sepsi

XML Treatment for Sepsis spura
